# Multi-omics integration reveals the role of N6-methyladenosine in epilepsy, ischemic stroke, and vascular dementia

**DOI:** 10.1186/s13041-025-01228-4

**Published:** 2025-07-07

**Authors:** Xudong Zhang, Yuhao Xu, Hai Hu, Zhenhua Liao, Changli Lou, Xiang Zou

**Affiliations:** 1 Department of Neurosurgery, Huashan Hospital, Fudan University, Shanghai, 200040 China; 2 Neurosurgery, Xingguo Hospital, Gannan Medical University, Ganzhou, China

**Keywords:** N6-methyladenosine (m6A), Epilepsy, Ischemic stroke, Vascular dementia (VaD), Multi-omics integration

## Abstract

**Background:**

N6-methyladenosine (m6A) methylation is an essential epigenetic modification that regulates mRNA stability, splicing, and translation. Its role in neurological diseases, including epilepsy, ischemic stroke, and vascular dementia (VaD), remains poorly understood.

**Methods:**

We integrated multi-omics data, including GWAS, m6A quantitative trait loci (QTL), expression QTL (eQTL), and protein QTL (pQTL), and using FUSION to assess the association of m6A with these diseases. Transcriptome-wide association studies (TWAS) and Mendelian Randomization (MR) were performed to identify causal relationships between m6A sites, gene expression, and disease. Differentially expressed genes (DEGs) were analyzed via RNA sequencing and enriched for biological pathways. Protein-protein interaction (PPI) networks and m6A-related gene-disease associations were constructed to reveal regulatory mechanisms.

**Results:**

We identified 218 m6A sites significantly associated with the three diseases, highlighting 3,430 associations between m6A sites and gene expression. Functional enrichment analysis revealed key pathways, including base excision repair and chemokine-mediated signaling. MR analysis identified causal relationships, such as NBL1 in epilepsy, TPGS2 in ischemic stroke, and SERINC2 in VaD. PPI analysis revealed interactions involving critical proteins like PARP1, MCL1, and CD40, underscoring their role in neuroinflammation and apoptosis.

**Conclusion:**

Our findings elucidate the genetic and epigenetic roles of m6A in epilepsy, ischemic stroke, and VaD, uncovering potential mechanisms by which m6A modulates gene and protein expression to influence disease outcomes. These insights highlight m6A as a promising biomarker and therapeutic target for neurological diseases.

**Supplementary Information:**

The online version contains supplementary material available at 10.1186/s13041-025-01228-4.

## Introduction

Epilepsy is a common neurological disorder affecting around 70 million people globally, with about 2.5 million new cases annually. Its prevalence ranges from 0.5 to 1% in developed countries to as high as 2% in developing regions, where nearly 80% of cases occur. Stigma and limited healthcare resources leave over 75% of those with active epilepsy untreated, especially in low- and middle-income countries [1]. Epilepsy rates are higher in children and the elderly, with older populations often developing epilepsy due to cerebrovascular disease. Ischemic stroke, accounting for 85% of all strokes, occurs when brain blood flow is obstructed, leading to tissue damage. Post-stroke epilepsy accounts for nearly 50% of newly diagnosed epilepsy annually, with higher prevalence in those over 65 and in individuals with conditions like cardiovascular disease and diabetes [2]. Stroke rates are also higher in low- and middle-income countries, reflecting disparities in healthcare access. VaD, the second most common form of dementia after Alzheimer’s, is caused by reduced blood flow to the brain, often due to strokes or vascular conditions like hypertension and atherosclerosis. Symptoms include memory loss, confusion, and impaired reasoning, with risk factors closely linked to cerebrovascular diseases like ischemic stroke [3].

Epilepsy, Ischemic stroke, and VaD often intertwine in elderly patients. Stroke can not only directly trigger epileptic seizures but also lead to VaD through cerebrovascular damage. At the same time, epileptic seizures may exacerbate the sequelae of stroke by further impairing vascular health or accelerating the progression of dementia. These conditions are often interrelated, forming a vicious cycle that accelerates the deterioration of the patient’s neurological function.

M6A is a prominent RNA modification in the brain, playing key roles in neuronal function and disease development [4]. In epilepsy, m6A modification is involved in regulating synaptic function, neural damage, cognitive impairment, and brain development abnormalities [5]. The m6A modification process is controlled by a complex of methyltransferases, demethylases, and binding proteins. Studies have shown that m6A-related enzymes, such as METTL3, FTO, and ALKBH5, influence epilepsy progression through their effects on gene expression linked to neuronal excitability, synaptic transmission, and oxidative stress. For instance, FTO downregulation promotes neuronal damage and exacerbates epilepsy progression, while METTL3 inhibition can reduce neuronal injury [6, 7]. In animal models, altered m6A levels impact memory and cognitive function, as seen with METTL3-mediated m6A, which enhances learning and memory [8]. Additionally, overexpressed FTO in neurons increases oxidative stress and Ca^2+^ influx, leading to neuron degeneration [9]. These findings underscore that m6A dysregulation contributes to synaptic dysfunction and impaired memory, which are critical aspects of epilepsy. Although current research is limited, targeting m6A modification holds potential for understanding and treating epilepsy and related cognitive impairments.

In ischemic stroke, m6A methylation has been shown to undergo significant alterations, indicating its potential role in the pathophysiology of the disease. Studies have observed elevated m6A levels in patients with ischemic stroke, as evidenced by increased global m6A levels in RNA from peripheral blood samples of stroke patients compared to healthy controls [10]. Animal models, particularly the middle cerebral artery occlusion (MCAO) model in rats and mice, have further demonstrated this effect. In these models, the peri-infarct cerebral cortex shows a marked rise in m6A methylation levels compared to sham controls, with immunofluorescence staining confirming this increase primarily in neurons [11]. The dynamic nature of m6A changes is evident during the ischemia and reperfusion phases. For instance, m6A levels increase transiently immediately after reperfusion, but subsequently decline at later time points. This pattern is consistent in both male and female mice subjected to MCAO, reinforcing the idea that stroke leads to a global upregulation of m6A methylation [12].

In VaD, m6A methylation impacts neuroinflammation, neuronal injury, and cerebrovascular function, all of which are key to the progression of the disease. VaD, primarily caused by impaired blood flow and resulting brain damage, is closely linked to neurovascular dysfunction and cognitive decline, areas where m6A modification is influential [13]. m6A regulates the expression of inflammatory cytokines like Interleukin 1 (IL-1) and Tumor Necrosis Factor Alpha (TNF-α), which are central to neuroinflammation, a major driver of VaD. By stabilizing or destabilizing the mRNA of these cytokines, m6A can modulate the inflammatory response, influencing the extent of neuronal damage and accelerating cognitive decline [14]. Additionally, m6A affects synaptic function and neuronal plasticity, crucial for learning and memory, as its dysregulation can lead to synaptic instability and neuron loss, further impairing cognitive function in VaD. In terms of cerebrovascular health, m6A influences blood-brain barrier integrity and endothelial cell function by modulating factors involved in angiogenesis and vascular stability [15]. This regulation affects blood flow and nutrient supply to the brain, potentially reducing the risk of blood-brain barrier breakdown, which is common in VaD [16]. Differences in m6A methylation patterns observed between VaD patients and healthy individuals suggest that m6A could serve as both a biomarker and therapeutic target, though further research is needed to clarify its complex interactions in the pathophysiology of VaD [17].

SNPs can influence m6A modification in much the same way they affect other regulatory marks—by altering the local RNA sequence context that is recognized by the methylation machinery. Many m6A “writer” complexes (e.g. METTL3/METTL14) preferentially methylate the consensus DRACH motif (D = A/G/U, R = A/G, H = A/C/U). A single-nucleotide change within or adjacent to this motif can create a new DRACH site, abolish an existing one, or modulate the surrounding secondary structure, thereby increasing or decreasing the likelihood that a given adenosine will be methylated. In population terms, these sequence variants manifest as m6A-quantitative trait loci (m6A-QTLs): SNPs whose alleles are statistically associated with differential m6A levels at a nearby site. By integrating m6A-QTL mapping with GWAS, we can therefore nominate disease-associated variants that exert their effects, at least in part, by rewiring the epitranscriptomic landscape—altering transcript stability, splicing, or translation via gain or loss of m6A marks. However, the genetic components and specific gene loci associated with m6A modifications in these diseases require further investigation. While m6A’s functional roles are increasingly understood, the underlying genetic loci and disease-specific molecular phenotypes that influence m6A modifications remain largely unexplored.

In this study, we aimed to uncover common epitranscriptomic regulators that transcend age and etiology, highlighting m6A as a unifying molecular node in neurovascular health and disease. We conducted an m6A association study (m6A-WAS) and Mendelian randomization (MR) analysis for three diseases (including epilepsy, ischemic stroke, and VaD) by integrating large-scale GWAS and human m6A QTL datasets. We then incorporated additional datasets from gene expression quantitative trait loci (eQTL) and protein quantitative trait loci (pQTL) to explore the complex interactions among these molecular phenotypes. This analysis includes the effects of m6A on gene expression, the influence of gene expression on m6A modification, and the post-transcriptional effects of m6A on protein levels. Notably, we focused on m6A sites significantly associated with the diseases to map causal relationships among these molecular phenotypes, as well as potential differential regulatory patterns between disease-associated SNPs identified through GWAS and m6A modification.

## Method

### Study design and data sources

**Fig. 1:**
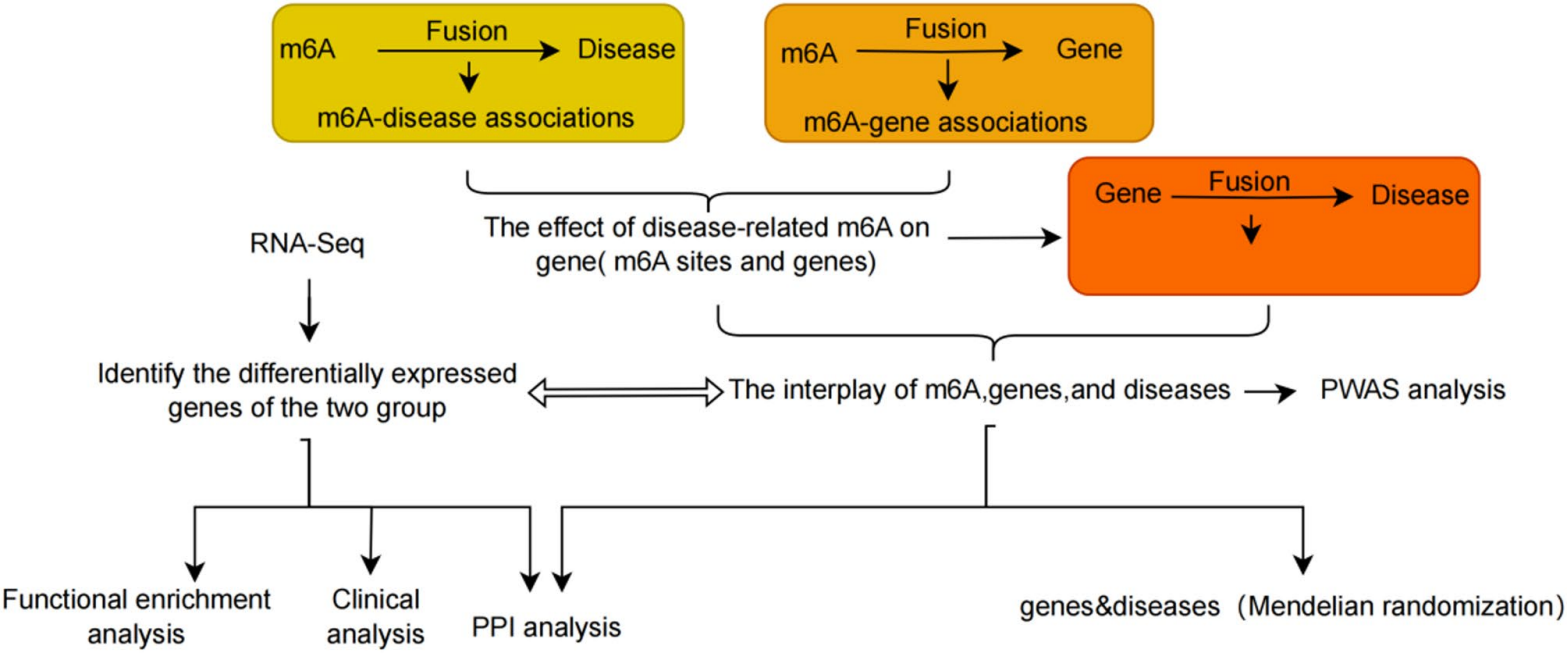
Flowchart of the analysis pipeline

The study design flowchart is presented in Fig. 1. This study utilized three large-scale GWAS summary datasets. We obtained ischemic stroke GWAS summary statistics from the GWAS Catalog database (https://www.ebi.ac.uk/gwas/), specifically dataset GCST006908, which includes 34,217 cases and 406,111 controls of European ancestry [18]. Additionally, VaD GWAS summary statistics were downloaded from the FinnGen database (https://r10.finngen.fi/), under dataset ID R10_F5_VASCDEM, consisting of 2,717 cases and 393,024 controls of European descent. We further acquired epilepsy GWAS summary statistics from the same database, using dataset ID R10_GE, comprising 1,413 cases and 399,287 controls of European ancestry. For further details on each GWAS dataset, please refer to the respective primary studies.

The blood eQTL data were derived from the eQTLGen database (https://eqtlgen.org/), containing cis-eQTLs for 16,989 genes identified from 31,684 blood samples of healthy individuals of European ancestry. Only significantly associated cis-eQTL results (false discovery rate (FDR) < 0.05) and allele frequency information were included in this analysis. m6A weight data were obtained from the study by Liufu et al. [19], with gene weights derived from the GTEx V8 database, which includes a whole-blood dataset of 670 samples analyzed using FUSION. The pQTL data were sourced from Wingo et al., comprising 376 brain tissue samples [20].

### Fusion analysis

To assess the genetic association between m6A methylation weights and disease traits (GWAS) or gene/protein levels (eQTL/pQTL), we used the FUSION.assoc_test.r function within the FUSION tool [19]. This approach enabled testing for associations between m6A loci and disease or gene expression/protein abundance. Similarly, we calculated genetic associations between gene/protein weights and disease traits to examine the relationship between gene/protein levels and disease outcomes.

### MR analysis of gene eQTL data and disease

We conducted MR analysis using the R package TwoSampleMR, selecting gene eQTL data as the exposure. To generate instrumental variables, we chose SNPs within 1 MB of each gene locus that met the FDR < 0.05 threshold. Linkage disequilibrium analysis was then performed on these SNPs using European samples from the 1000 Genomes Project, setting an r² threshold < 0.01 and kb = 10,000. A critical step in MR analysis is to ensure that the effect of each SNP on the exposure corresponds to its effect on the outcome with respect to the same allele. To maintain consistency in effect allele orientation, we removed palindromic SNPs (e.g., those with A/T or G/C alleles). The selection of these instrumental variables ensures the reliability of our findings. We then performed MR analysis to evaluate the causal relationship between gene expression and outcomes, identifying potential target genes. The Wald ratio method was used to calculate the MR estimate for each SNP. When multiple SNPs were available, we applied a weighted mean of the ratio estimates, using inverse variance weighting for weighting the estimates. For analyses involving three or more SNPs, we evaluated the MR-Egger intercept to check if it significantly deviated from zero, testing for horizontal pleiotropy. Additionally, Cochran’s Q test was used to assess heterogeneity among the Wald ratios. The I² statistic was calculated based on the Q value to estimate the proportion of total variation attributable to heterogeneity rather than sampling error, using the formula I^2^ = (Q-Q_df_) / Q. We also conducted a leave-one-out sensitivity analysis to determine whether any significant associations were driven by a single SNP, which involved iteratively performing MR by excluding one SNP at a time. All these analyses, including sensitivity and MR analyses, were carried out in the TwoSampleMR package in R [21].

### Enrichment analysis

Gene Ontology (GO) analysis, a primary bioinformatics tool for annotating genes and their products, categorizes annotations into three main classes: Cellular Component (CC), Molecular Function (MF), and Biological Process (BP). Kyoto Encyclopedia of Genes and Genomes (KEGG) provides a comprehensive collection of databases containing information on genomes, biological pathways, diseases, and chemical substances. We utilized the enrichGO and enrichKEGG functions in the clusterProfiler package to perform GO and KEGG pathway analyses, predicting potential molecular functions of the identified genes [22]. For these analyses, the parameters used were pvalueCutoff = 0.05, pAdjustMethod = “BH”, and qvalueCutoff = 0.5. PPI data were obtained from the STRING database (https://string-db.org/), which allowed us to construct a protein interaction network to visualize and explore the relationships among the proteins encoded by our genes of interest.

### PWAS between m6A-related genes and target proteins

We performed a proteome-wide association study (PWAS) using the Ontime database developed by Professor Ting Wang’s laboratory at Washington University School of Medicine in St. Louis (https://ontime.wustl.edu/hg19/top_hits*).*

### Transcriptomic data analysis

Transcriptomic data for epilepsy (GSE163296), consisting of 22 samples, was downloaded from the GEO database [23]. The expression data were processed as follows: (1) empty probes were removed; (2) probes mapping to multiple genes were deleted; (3) for genes with multiple corresponding probes, the median expression value of these probes was used to represent the gene’s expression level.

Differentially expressed genes (DEGs) between the disease and control samples were identified using the limma package in R [24]. Genes were considered significantly differentially expressed based on the thresholds of log2 fold change (log2FC) ≥ 0.585 and *P* < 0.05.

### Construction of PPI network and identification of hub genes

PPIs form a crucial part of cellular biochemical reaction networks. Evaluating PPI networks and understanding their functions are key to gaining insights into cellular mechanisms within cellular and molecular systems biology. To construct the PPI network, we used the STRING database (https://cn.string-db.org/) with the following parameters: disconnected nodes were hidden in the network display options [25]. To capture a more comprehensive set of protein interactions and generate a more intricate network, we set the minimum required interaction score to a moderate confidence level of 0.4. Network topology analysis was conducted using the Cytoscape plugin “cytoHubba,” selecting the top 50 nodes based on their Degree ranking to identify key hub genes.

### Statistical analysis

Briefly, our GWAS cohorts comprised 34,217 ischemic stroke cases versus 406,111 controls, 2,717 vascular dementia cases versus 393,024 controls, and 1,413 epilepsy cases versus 399,287 controls. The blood eQTLGen dataset includes 31,684 healthy donors, the GTEx-derived m6A weight panel uses 670 whole-blood samples, and the brain pQTL resource comprises 376 post-mortem samples. For transcriptomic profiling (GSE163296), we analyzed 22 surgical hippocampus samples (11 epilepsy patients and 11 age- and sex-matched controls). We performed association analysis to examine the relationship between the expression levels of m6A-related genes and proteins in the transcriptome and the clinical and pathological characteristics of epilepsy patients. All statistical analyses were conducted using R software (version 4.1.2; https://www.R-project.org) [26]. Heatmaps and volcano plots of DEGs were generated using the pheatmap and ggplot2 packages, respectively. A p-value of less than 0.05 was considered statistically significant for all analyses. By default, significance levels are indicated as follows: *** for *p* < 0.001, ** for *p* < 0.01, * for *p* < 0.05, and ns for non-significant results.

## Results

### Disease GWAS data information

**Fig. 2:**
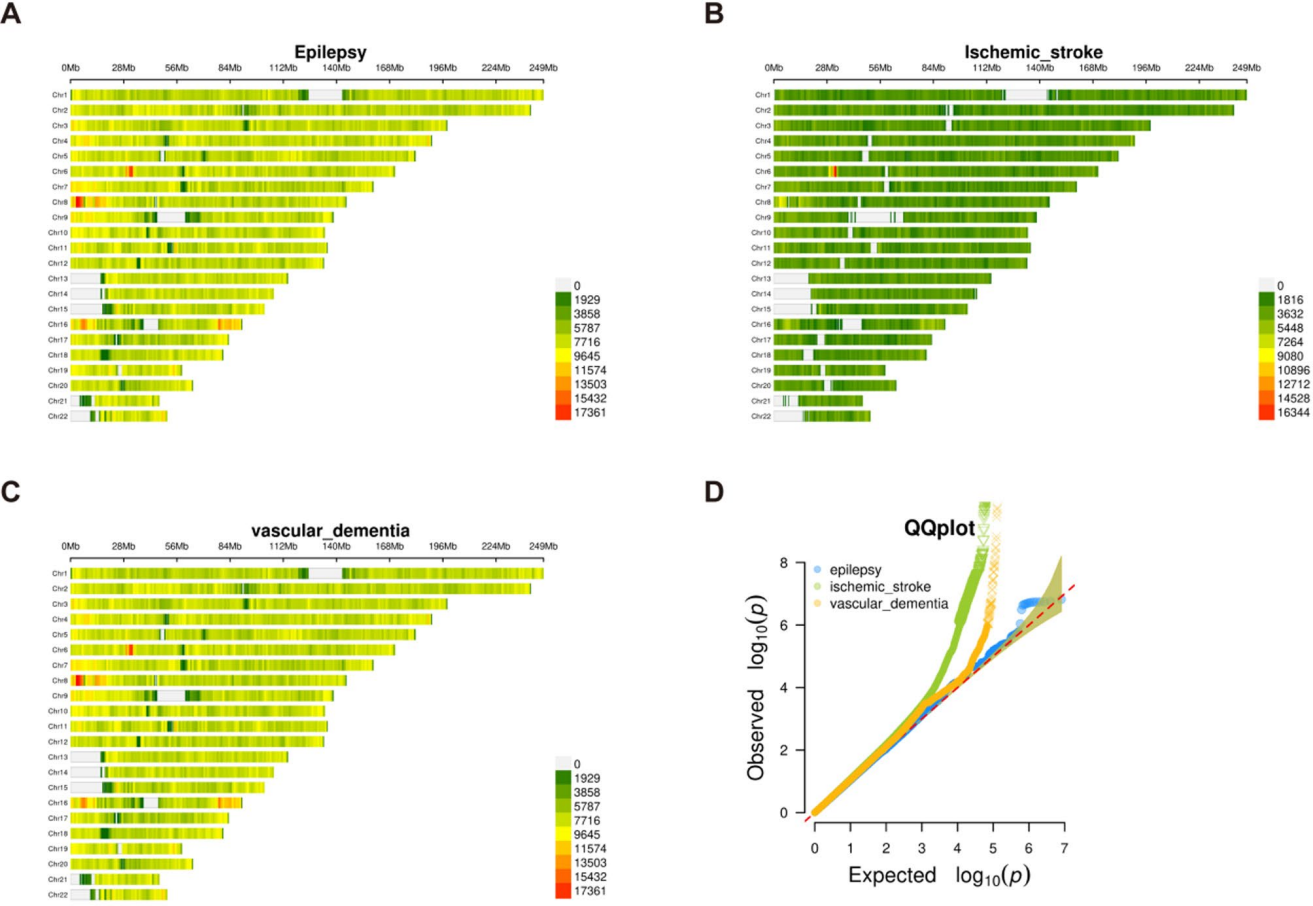
GWAS Data Information. **A**: SNP density plot of GWAS for epilepsy. **B**: SNP density plot of GWAS for ischemic stroke. **C**: SNP density plot of GWAS for VaD. **D**: QQ plot of GWAS for the diseases

We examined GWAS data for epilepsy, ischemic stroke, and VaD. Figure 2A-C shows the SNP density information for each condition, indicating that the number of SNPs for epilepsy and VaD is significantly higher than for ischemic stroke. Figure 2D displays Quantile-Quantile (QQ) plots for the three diseases, with a notable upward deviation of points, potentially suggesting the presence of genuine association signals in the GWAS results.

### Association between m6A, genes and diseases

**Fig. 3:**
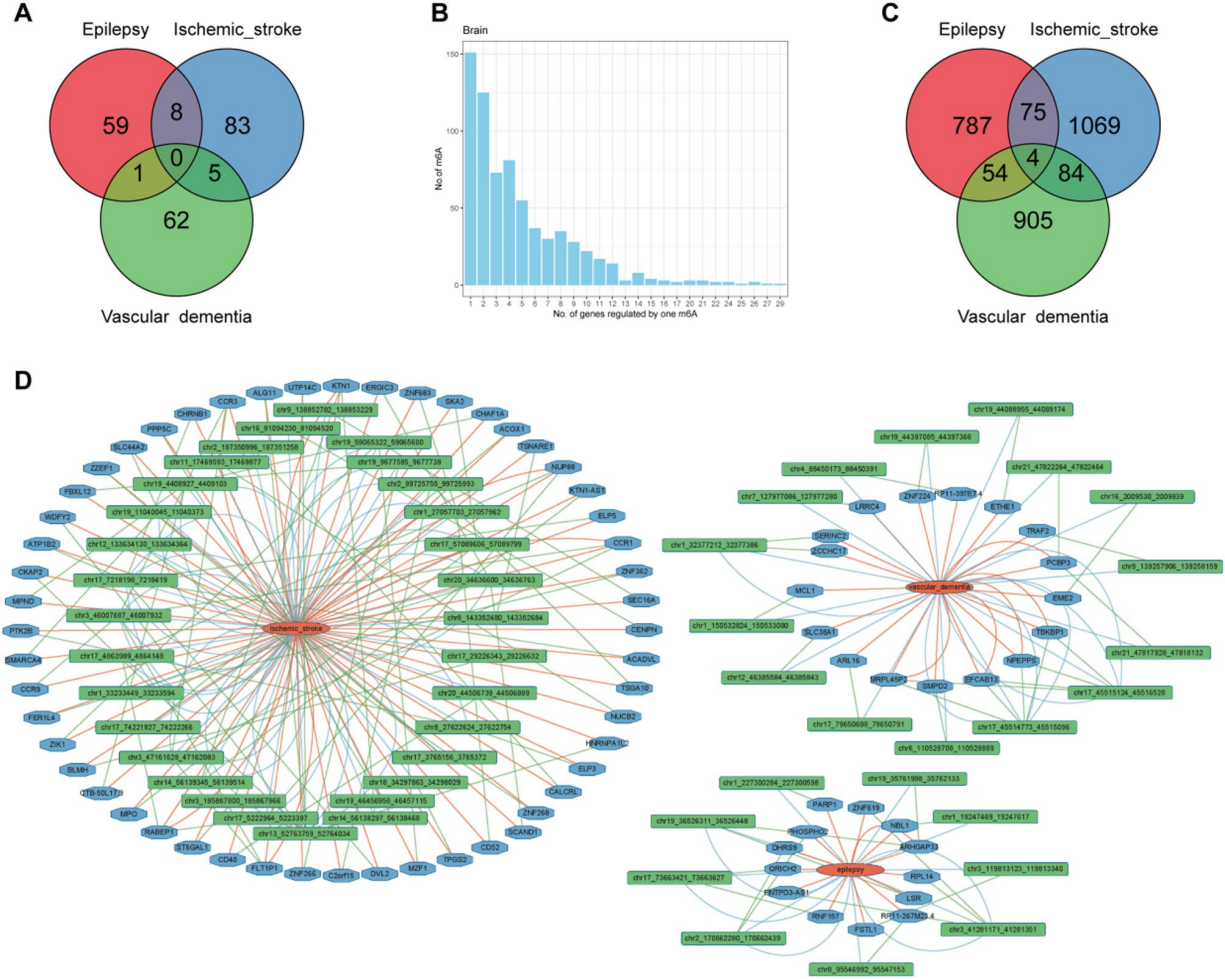
Association Between m6A, Genes and Diseases. **A**: The Venn diagram shows the overlap of m6A sites associated with epilepsy, ischemic stroke, and VaD. **B**: The histogram illustrates the distribution of m6A sites in the brain according to the number of genes regulated by each site. A majority of m6A sites regulate one or two genes, with a decreasing frequency of m6A sites as the number of regulated genes increases. **C**: Venn Diagram of Genes Associated with Epilepsy, Ischemic Stroke, and VaD. **D**: Interaction Network of m6A, Gene Expression, and Disease. The interaction network illustrates the relationships among m6A sites, gene expression, and disease. Red ellipses represent diseases, blue octagons denote genes, and green triangles indicate m6A sites. Red lines signify associations between diseases and genes, green lines represent associations between m6A sites and genes, and blue lines depict associations between m6A sites and diseases

To identify m6A sites associated with disease, we used the FUSION pipeline to calculate the association between brain m6A weights and three diseases: epilepsy, ischemic stroke, and VaD. We detected 232 significant associations involving 218 m6A sites across the three diseases (Table S1), with the majority related to ischemic stroke (96 associations), followed by epilepsy and VaD (68 associations each). Eight m6A sites were shared between epilepsy and ischemic stroke: chr1_118321088_118321459, chr10_126524158_126524410, chr12_108919143_108919365, chr16_81094230_81094520, chr19_13001948_13002160, chr2_166848125_166848454, chr3_47161628_47162083, and chr8_68069943_68070153. One m6A site was common between epilepsy and VaD: chr3_105377827_105378037. Five m6A sites were shared between ischemic stroke and VaD: chr10_135370501_135370600, chr3_50712328_50712720, chr3_51198350_51198948, chr3_69082604_69082847, and chr4_88450173_88450391 (Fig. 3A).

To elucidate the association between m6A and gene expression, we employed the FUSION method to evaluate this relationship. Utilizing m6A weights and the eQTLGen summary dataset, we identified 3,430 significant associations, including 790 m6A sites and 2,457 genes, which passed the multiple testing correction threshold (Table S2). The threshold was calculated as *P* < 0.05 / (N_probes × N_genes), where N_probes represents the number of probes in the weights and N_genes refers to the number of genes in the eQTL dataset. Our results demonstrate that the majority of m6A sites influence multiple genes, with an average of 3.11 genes affected by a single m6A site in brain tissue (Fig. 3B).

To identify gene loci associated with diseases, we utilized the FUSION pipeline to calculate gene weights in relation to three conditions: epilepsy, ischemic stroke, and VaD. We detected 3,979 genes with a total of 3,224 significant associations across the three diseases (Table S3). Most of these associations were related to ischemic stroke, with 1,242 associations; followed by epilepsy with 928 associations, and VaD with 1,054 associations. Among these, 79 genes were shared between epilepsy and ischemic stroke, 59 genes between epilepsy and VaD, and 88 genes between ischemic stroke and VaD. Notably, there were four genes common to all three diseases: PMF1, USP5, DYNC2LI1, and CENPW (Fig. 3C).

### The interaction of m6A, genes, and diseases

#### Identification of associated genes

By integrating the associations among m6A, gene expression, and diseases, we can potentially identify genes and regulatory elements relevant to disease functions. We combined the associations of m6A-disease, m6A-gene, and gene-disease (Fig. 3D, Table S4). In epilepsy, this included 13 genes and 9 m6A sites; ischemic stroke included 52 genes and 31 m6A sites; and VaD included 17 genes and 15 m6A sites. This integration reveals a network pattern of “m6A-gene-disease,” indicating that m6A regulates gene expression, which subsequently influences disease outcomes. For example, the m6A site chr1_32377212_32377386 is associated with VaD and the gene SERINC2, which is linked to VaD. Notably, this m6A site is also associated with ZCCHC17, highlighting its potential broader regulatory role.

### Functional analysis of associated genes

**Fig. 4:**
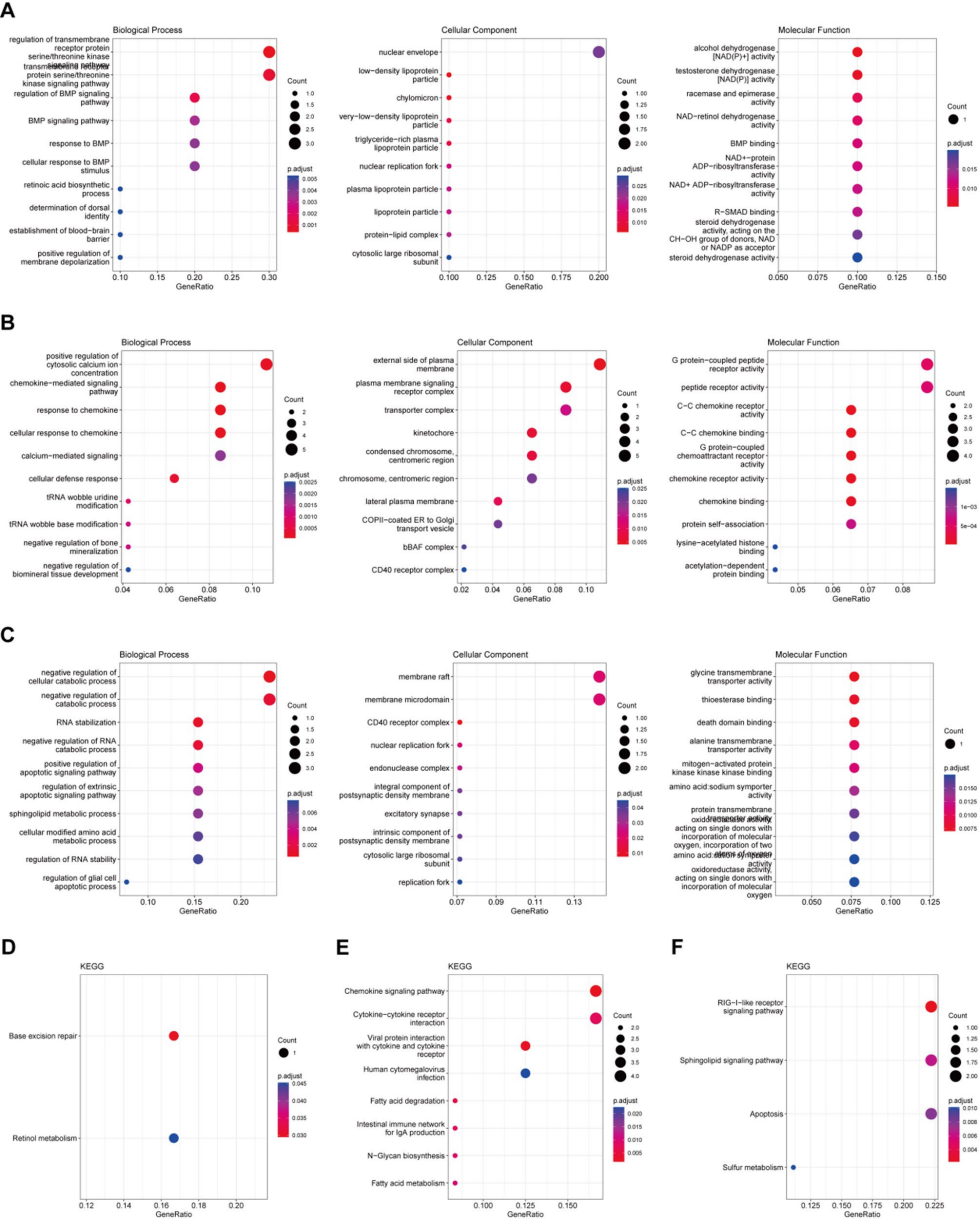
GO and KEGG Enrichment Analysis. GO enrichment analysis of genes associated with m6A and disease in epilepsy (**A**), Ischemic Stroke (**B**), and VaD (**C**). KEGG enrichment analysis of genes associated with m6A and disease in epilepsy (**D**), Ischemic Stroke (**E**), and VaD (**F**)

**Fig. 5:**
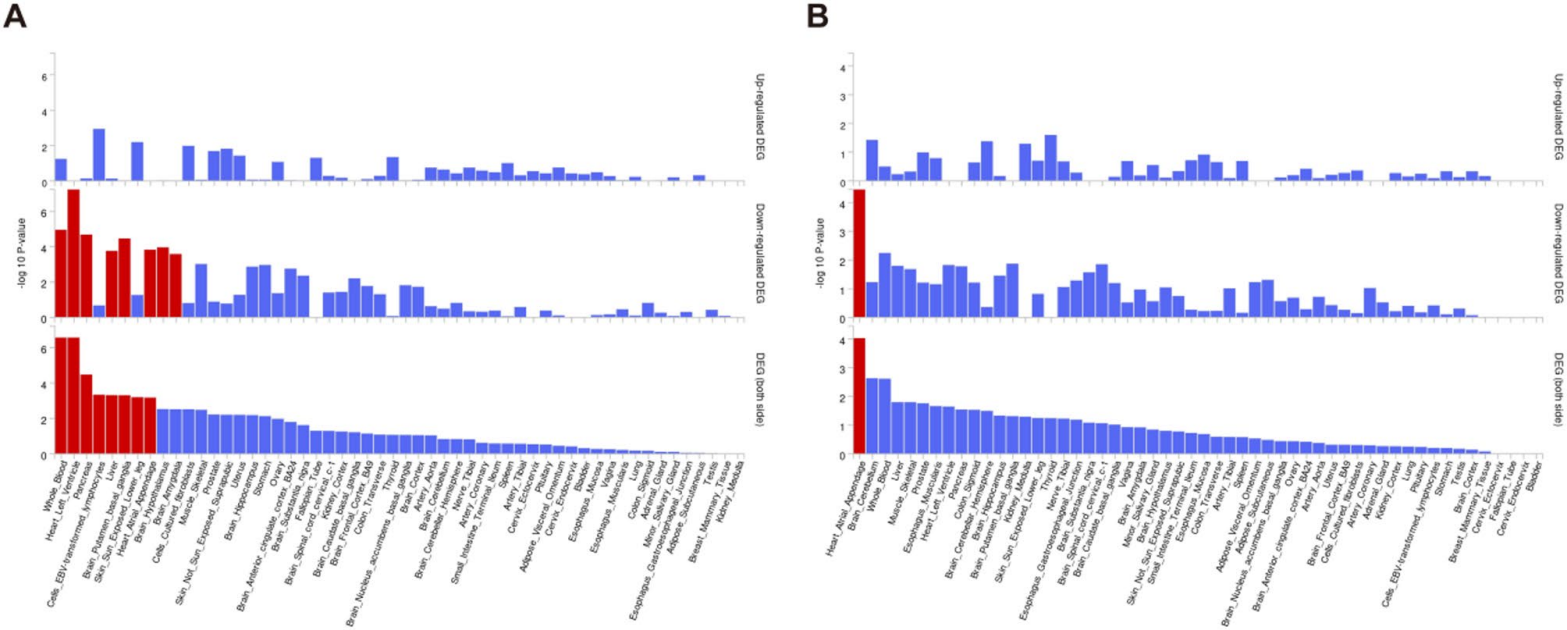
Tissue-specific enrichment analysis of ischemic stroke and vascular dementia. Using all genes obtained from the m6A gene-ischemic stroke (**A**) and gene-VaD (**B**) analysis, we performed tissue-specific enrichment with GENE2FUNC in FUMA, using all genes as the background. The enrichment analysis was conducted across 54 tissue types from GTEx v8. Tissues with significant enrichment (PFDR < 0.05) are highlighted in red

We performed enrichment analysis on the genes associated with each disease in the m6A-gene-disease framework. For epilepsy, the associated genes primarily involved the establishment of the blood-brain barrier and base excision repair pathways (Fig. 4A and D). In the case of ischemic stroke, the associated genes were mainly linked to cellular defense responses and chemokine-mediated signaling pathways (Fig. 4B and E). For VaD, the associated genes were primarily related to RNA stability, negative regulation of cellular catabolism, and apoptosis (Fig. 4C and F). Using the GENE2FUNC feature in FUMA, we conducted a tissue-specific analysis of the associated genes. We found that genes related to ischemic stroke were significantly enriched for downregulated genes in whole blood, heart, pancreas, liver, and brain (Fig. 5A). In contrast, when analyzing genes associated with VaD, we observed significant enrichment for downregulated genes specifically in whole blood (Fig. 5B).

### Causal association of associated genes with diseases

Using the plasma cis-eQTL data from the eQTLGene database (FDR < 0.05), we employed genes involved in the m6A-gene-disease framework for MR analysis. We screened for instrumental variables for each gene using linkage disequilibrium analysis (linkage disequilibrium parameter r² = 0.01, genetic distance = 10,000 kb). Each gene’s eQTL data related to the diseases was analyzed in a one-to-one MR framework (Table S5).

**Fig. 6:**
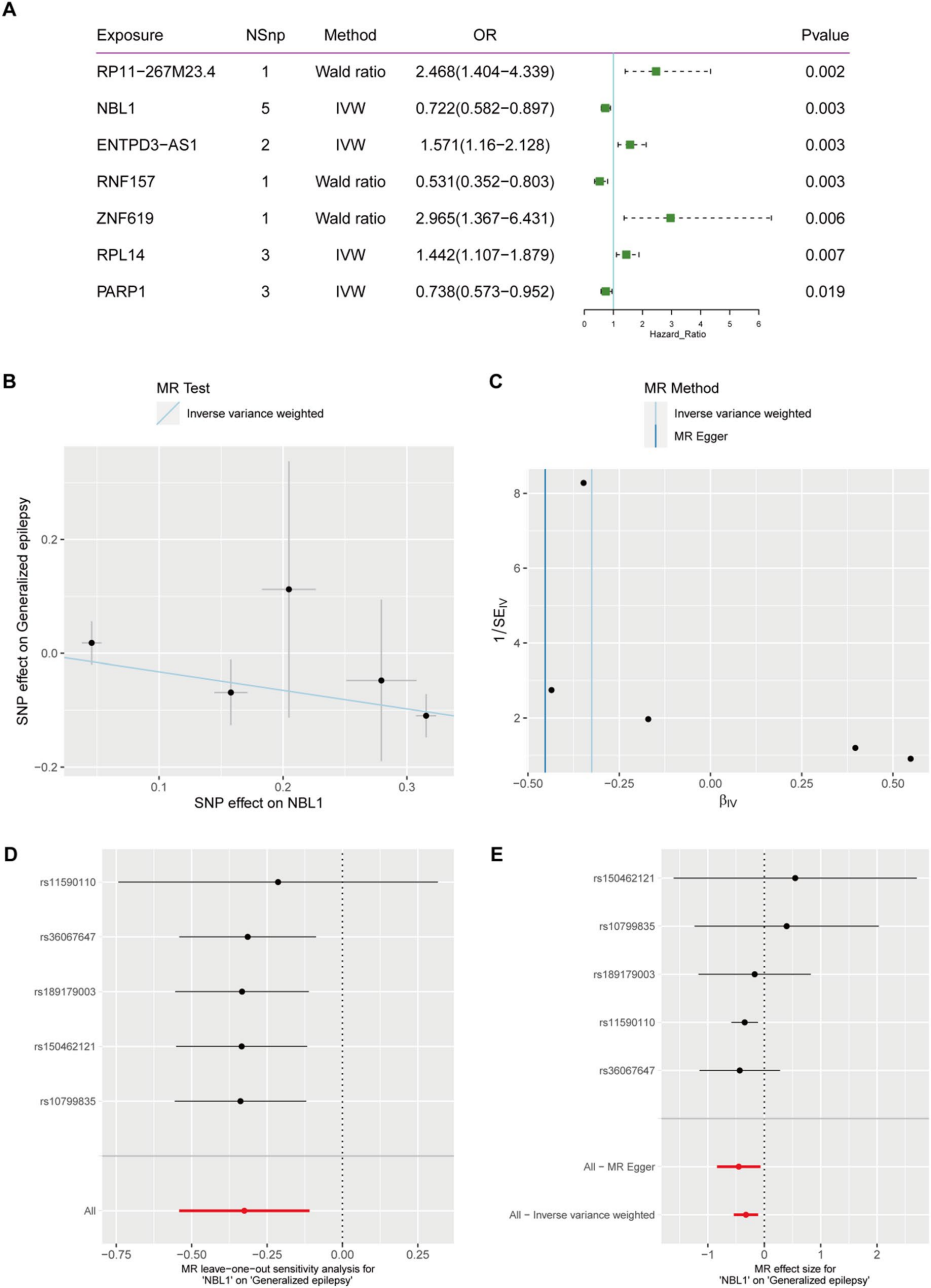
MR Analysis of eQTL, NBL1 Gene and Epilepsy GWAS Data **A**: This forest plot shows the MR analysis results of gene eQTL and GWAS data associated with epilepsy. The columns display the exposure genes, number of SNPs, MR methods (Wald ratio or IVW), odds ratio (OR) with confidence intervals, and p-values. Genes with significant associations are highlighted, where the OR indicates the direction and magnitude of the effect on epilepsy risk. **B**: Scatter plot of SNP effects on NBL1 and generalized epilepsy with IVW regression line. **C**: Funnel plot using IVW and MR Egger methods, assessing heterogeneity. **D**: Leave-one-out sensitivity analysis, showing robustness of results. **E**: Forest plot of SNP effect sizes on epilepsy with MR Egger and IVW methods

In the MR analysis with epilepsy as the outcome, we identified seven positive results: RP11-267M23.4, NBL1, ENTPD3-AS1, RNF157, ZNF619, RPL14, and PARP1 (Fig. 6A). The IVW model indicated a significant causal relationship between NBL1 and the epilepsy outcome (OR = 0.722, 95% CI = 0.582–0.897, *p* = 0.003, Fig. 6B), suggesting that an increase in the exposure factor is causally linked to a decrease in the outcome measure. Cochran’s Q test results showed no evidence of heterogeneity (Fig. 6C). Leave-one-out analysis indicated that the overall relationship estimate was not significantly affected by any individual SNP (Fig. 6D). Additionally, single SNP analysis revealed that certain SNPs exhibited a protective trend towards the outcome measure (Fig. 6E).

**Fig. 7:**
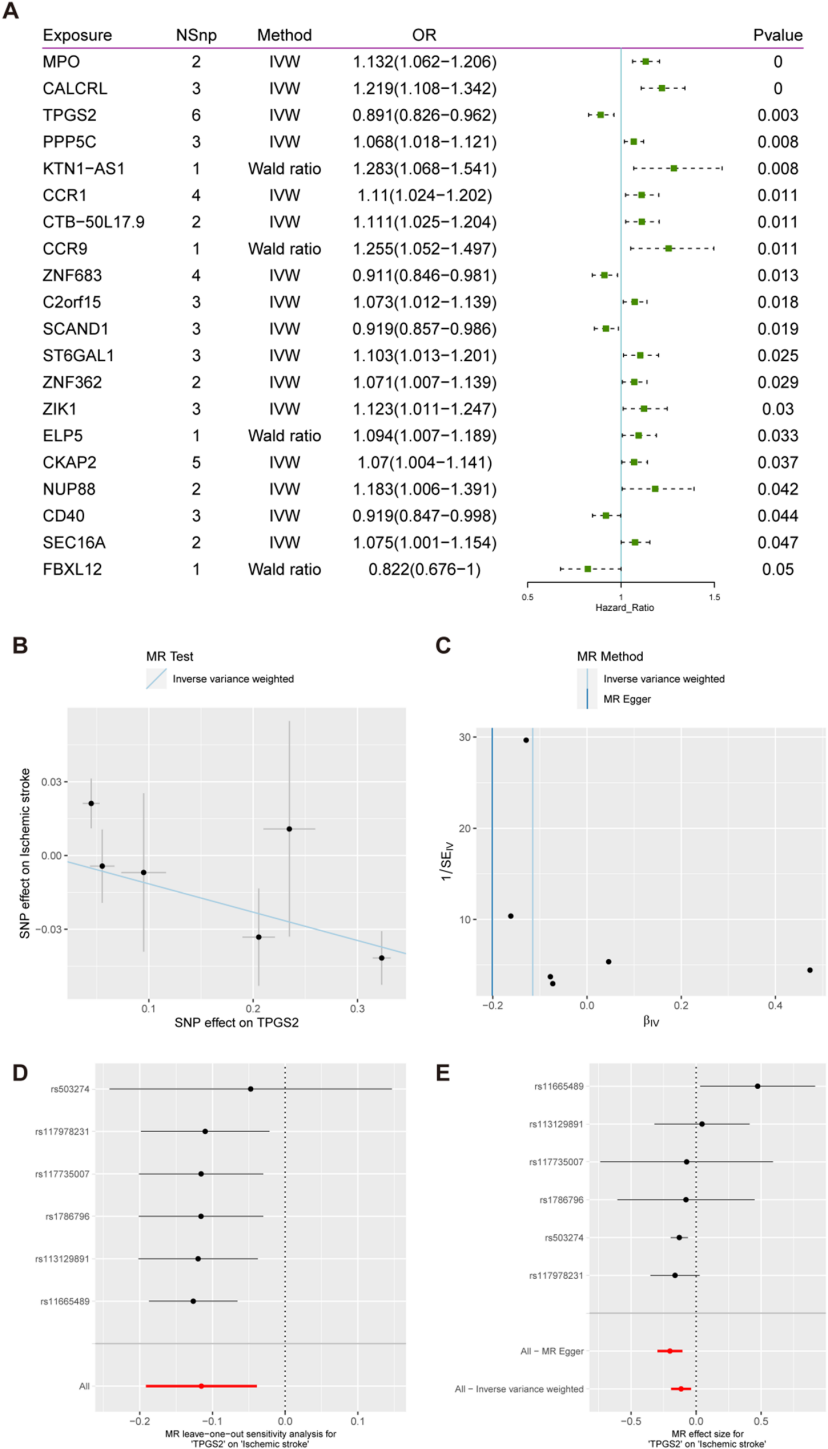
MR Analysis of eQTL, TPGS2 Gene and Ischemic Stroke GWAS Data. **A**: This forest plot shows the MR analysis results of gene eQTL and GWAS data associated with ischemic stroke. The columns display the exposure genes, number of SNPs (NSnp), MR methods (Wald ratio or IVW), odds ratio (OR) with confidence intervals, and p-values. Genes with significant associations are highlighted, where the OR indicates the direction and magnitude of the effect on Ischemic stroke risk. **B**: Scatter plot of SNP effects on TPGS2 and generalized ischemic stroke with IVW regression line. **C**: Funnel plot using IVW and MR Egger methods, assessing heterogeneity. **D**: Leave-one-out sensitivity analysis, showing robustness of results. **E**: Forest plot of SNP effect sizes on ischemic stroke with MR Egger and IVW methods

In the MR analysis with ischemic stroke as the outcome, we identified 20 positive results, including myeloperoxidase (MPO), CALCRL, and TPGS2 (Fig. 7A). The IVW model results indicated a significant causal relationship between TPGS2 and ischemic stroke (OR = 0.891, 95% CI = 0.826–0.962, *p* = 0.003, Fig. 7B), suggesting that an increase in the exposure factor is causally linked to a decrease in the outcome measure. Cochran’s Q test results showed no evidence of heterogeneity (Fig. 7C). Leave-one-out analysis indicated that the overall relationship estimate was not significantly influenced by any individual SNP (Fig. 7D). Furthermore, single SNP analysis revealed that certain SNPs exhibited a protective trend towards the outcome measure (Fig. 7E).

**Fig. 8:**
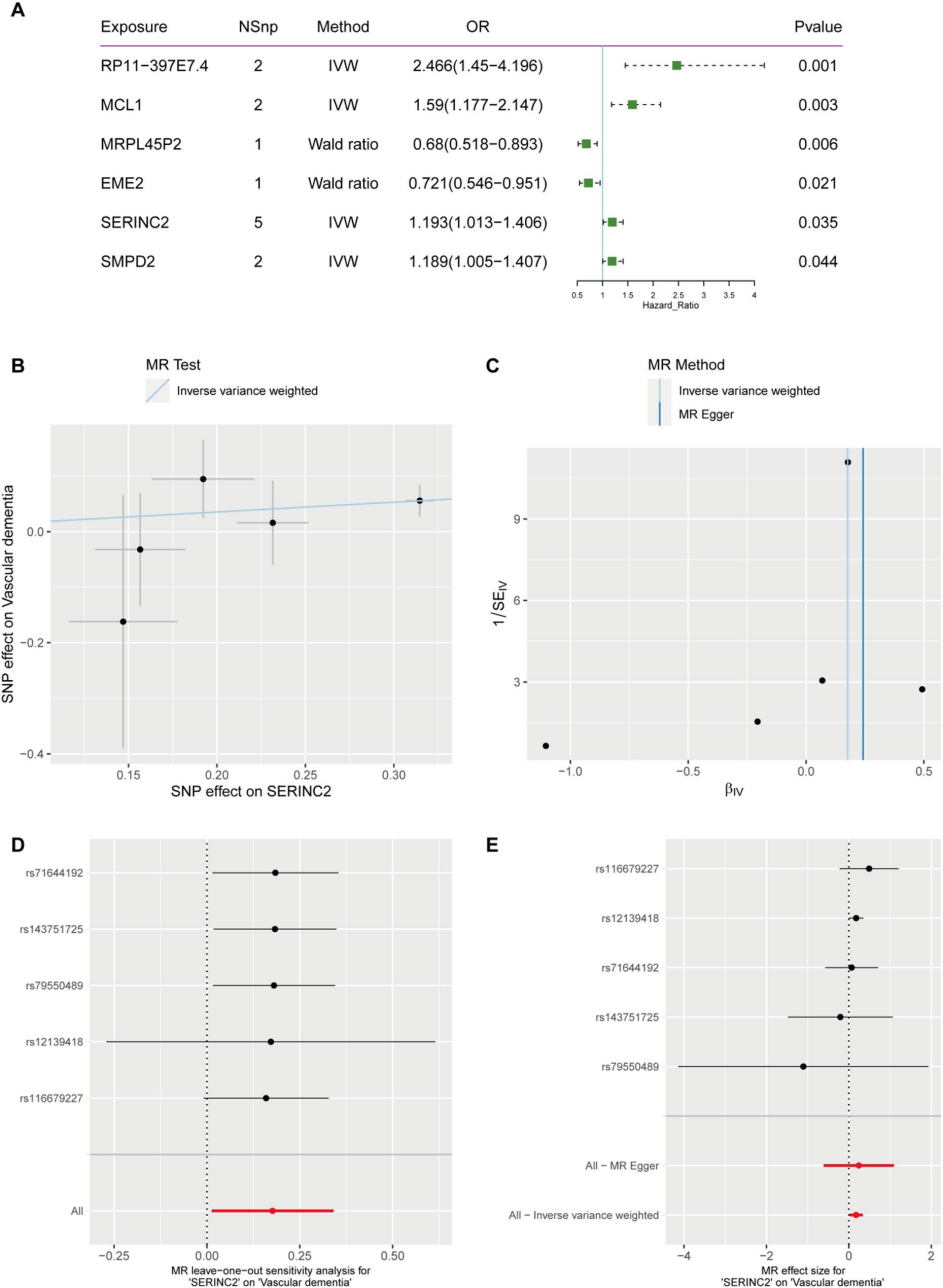
MR Analysis of eQTL, SERINC2 Gene and VaD GWAS Data. **A**: This forest plot shows the MR analysis results of gene eQTL and GWAS data associated with VaD. The columns display the exposure genes, NSnp, MR methods (Wald ratio or IVW), odds ratio (OR) with confidence intervals, and p-values. Genes with significant associations are highlighted, where the OR indicates the direction and magnitude of the effect on VaD risk. **B**: Scatter plot of SNP effects on SERINC2 and generalized VaD with IVW regression line. **C**: Funnel plot using IVW and MR Egger methods, assessing heterogeneity. **D**: Leave-one-out sensitivity analysis, showing robustness of results. **E**: Forest plot of SNP effect sizes on VaD with MR Egger and IVW methods

In the MR analysis with VaD as the outcome, we identified six positive results: RP11-397E7.4, MCL1, MRPL45P2, EME2, SERINC2, and SMPD2 (Fig. 8A). The IVW model results indicated a significant causal relationship between SERINC2 and VaD (OR = 1.193, 95% CI = 1.013–1.406, *p* = 0.035, Fig. 8B), suggesting that an increase in the exposure factor is causally linked to an increase in the outcome measure. Cochran’s Q test results indicated the presence of heterogeneity (Fig. 8C). Leave-one-out analysis showed that the overall relationship estimate was not significantly affected by any individual SNP (Fig. 8D). Additionally, single SNP analysis revealed that certain SNPs exhibited a protective trend towards the outcome measure (Fig. 8E).

### Interaction network of associated genes

**Fig. 9:**
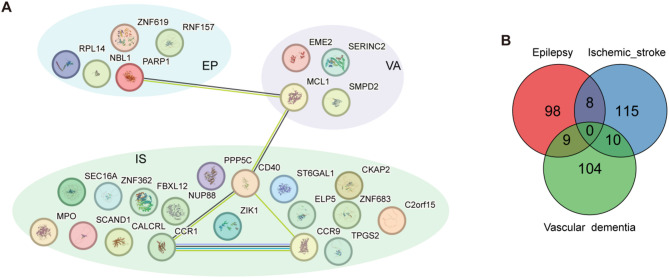
M6A, Disease and Genes. **A**: This PPI network illustrates the relationships among proteins associated with epilepsy (EP), VaD (VA), and ischemic stroke (IS). Each node represents a protein, with unique color-coded clusters indicating disease-specific associations: blue for epilepsy, purple for VaD, and green for ischemic stroke. Green lines indicate co-mentioned in Pubmed abstracts. Black lines indicate co-expression. Purple lines indicate protein homology. Blue lines indicate proteins from curated databases. **B**: The Venn diagram shows the overlap of proteins associated with epilepsy, ischemic stroke, and VaD

We selected genes from the m6A-gene-disease associations that exhibited causal relationships in the MR analysis for PPI network analysis. Notably, ELP5 showed inconsistent causal effect directions between the MR and FUSION analyses for ischemic stroke. Using the STRING database (https://cn.string-db.org/), we input the selected genes with a confidence score of 0.4, keeping all other parameters at default settings to construct the protein interaction network (Fig. 9A). The analysis revealed multiple protein-protein interactions of interest. PARP1 and MCL1 were found to exhibit co-expression and were also co-mentioned in PubMed abstracts, suggesting a functional and literature-based link. Similarly, MCL1 and CD40 showed both co-expression and frequent co-mentioning in PubMed abstracts, indicating their potential involvement in shared biological pathways. Furthermore, C-C Chemokine Receptor Type 1 (CCR1) and CD40 were identified as being co-expressed and co-mentioned in PubMed abstracts, highlighting another significant connection. Notably, CCR1 and C-C Chemokine Receptor Type 9 (CCR9) demonstrated the strongest relationship, being associated through co-expression, co-mentioning in PubMed abstracts, curated database associations, and protein homology, pointing to a robust interaction across multiple evidence layers.

### Association between proteins and diseases

To further investigate the association between genes and diseases, we conducted a FUSION analysis using pQTL data and GWAS. We identified 344 proteins with a total of 371 significant associations across the three diseases (Table S6). Most associations were related to ischemic stroke, with 133 associations, followed by epilepsy with 115 associations, and VaD with 123 associations. There were 8 proteins shared between epilepsy and ischemic stroke: SH3BGRL3, BPNT1, PTPMT1, PACSIN3, MIPEP, CMC2, ABCA5, and PTK2B. Additionally, 9 proteins were common to epilepsy and VaD: DOCK7, BCAN, RNPEP, REXO2, LRP4, IQGAP1, CES1, FER, and FAM131B. Furthermore, 10 proteins were shared between ischemic stroke and VaD: MTHFR, CISD1, UVRAG, HMBS, STAT6, IGLON5, FARP2, ICA1L, MON1A, and GRAMD3 (Fig. 9B).

### PWAS identification of m6A-related genes and target proteins in epilepsy

Based on previously identified m6A-related genes associated with epilepsy—ARHGAP33, DHRS9, ENTPD3-AS1, FSTL1, LSR, NBL1, PARP1, PHOSPHO2, QRICH2, RNF157, RP11-267M23.4, RPL14, and ZNF619—we performed online analyses using the comprehensive proteomic research cohort resources. This analysis identified the target proteins that have causal relationships with the m6A-target genes: ST6GALNAC5, CTNNA2, TOM1L1, CELSR2, and QRICH2.

### PPI network construction for identifying m6A-related proteins in cognitive impairment mechanisms

**Fig. 10:**
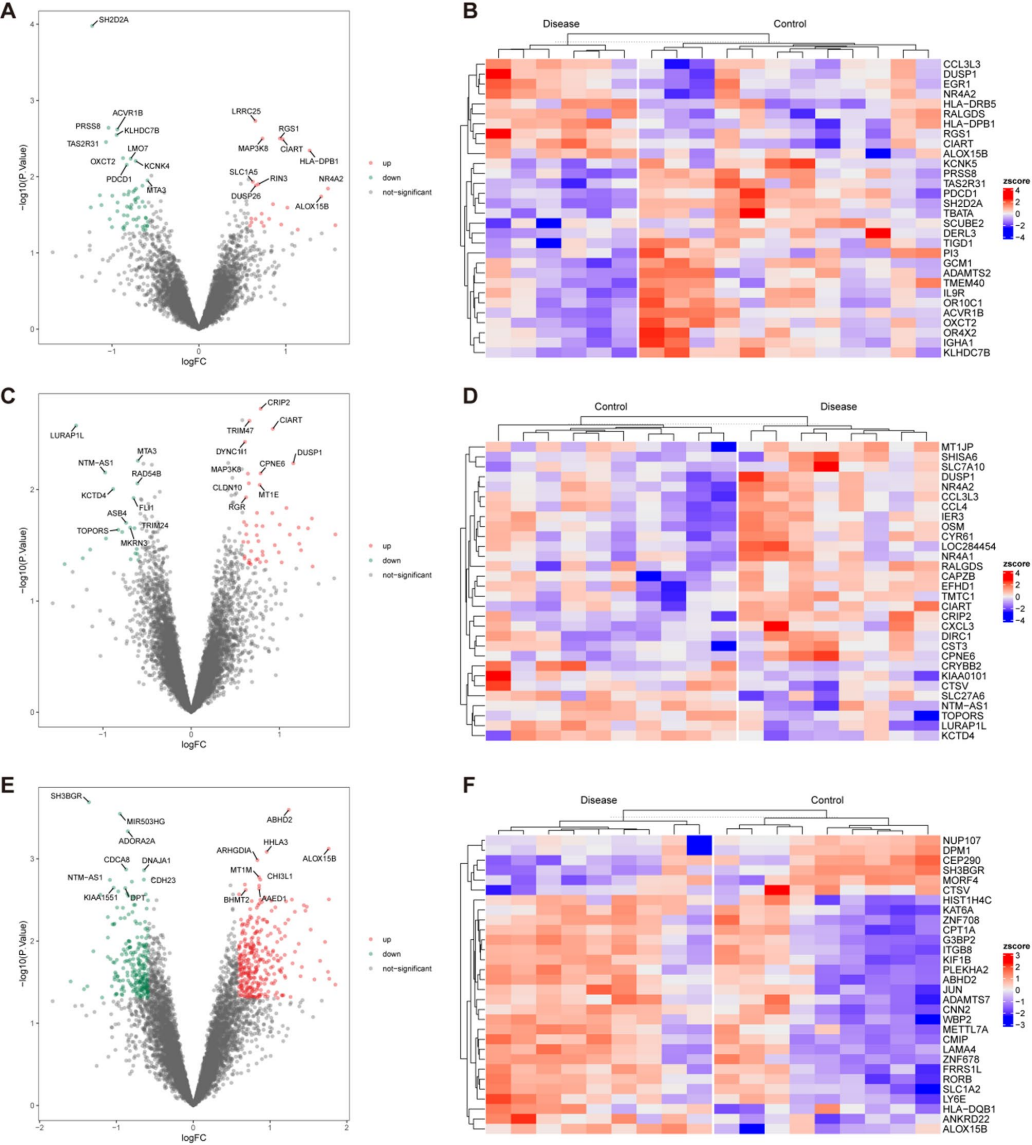
Differential Expression Analysis in the Language Impairment, Memory Impairment and Executive Function Impairments Group. The volcano plot of DEGs in the language impairment (**A**), memory impairment (**C**) and executive function impairments (**E**) group. The heatmap of the top 30 DEGs, with expression levels compared between language impairment (**B**), memory impairment (**D**) and executive function impairments (**F**) groups and control groups

**Fig. 11:**
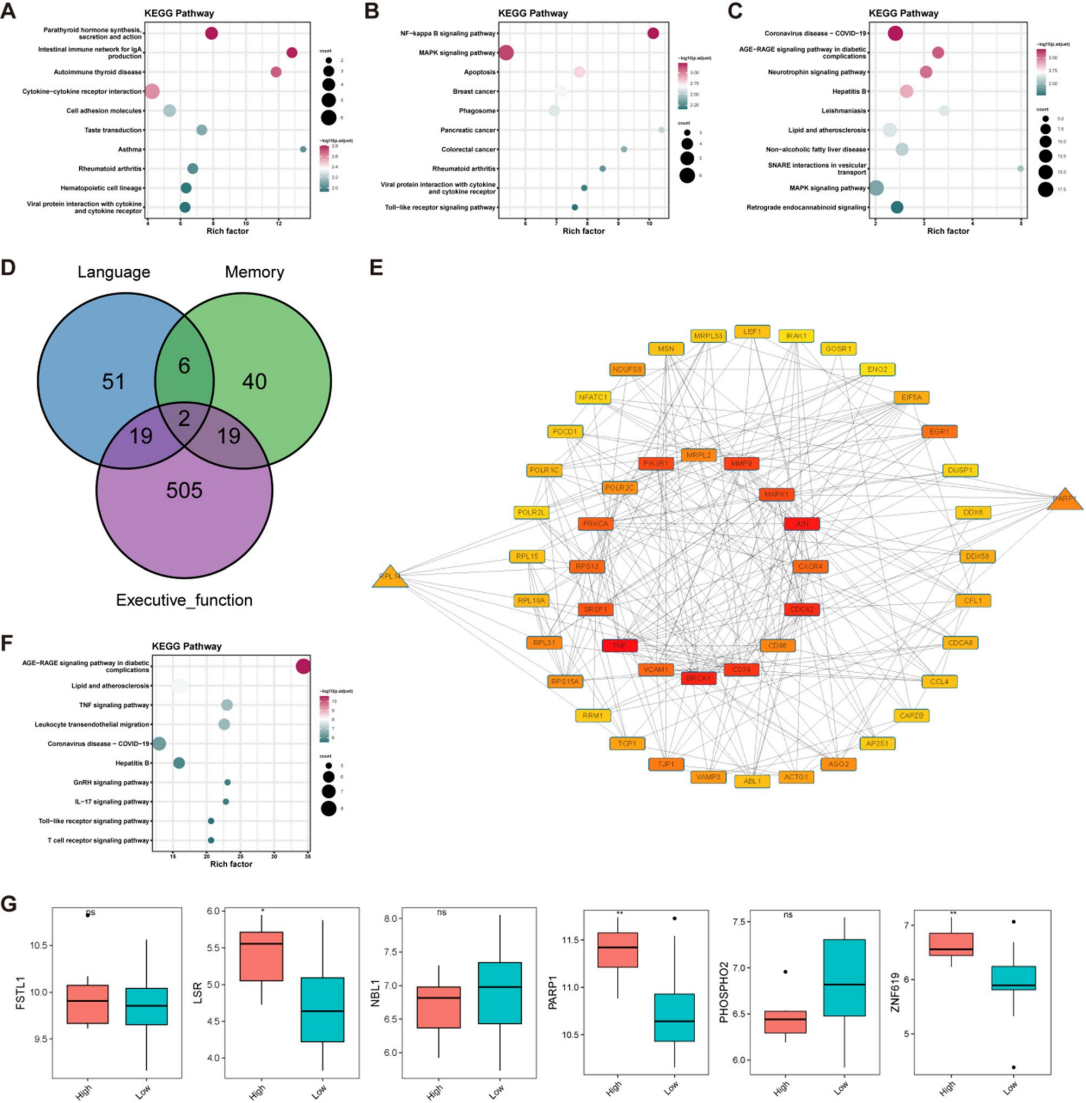
Clinical Correlation Analysis. KEGG Enrichment Results for DEGs of Language (**A**), Memory (**B**), and Executive Function Impairments (**C**) Groups. **D**: The Venn diagram shows the overlap of DEGs associated with language, memory, and executive function impairments. **E**: The PPI network illustrates the connections between m6A-related genes (indicated by triangles) and other DEGs, highlighting potential interactions within the network. Nodes in red represent highly interconnected genes. **F**: KEGG pathway enrichment analysis of key genes in the PPI network, showing enriched pathways associated with the DEGs. **G**: Expression of m6A-Related Genes in Epilepsy Patients Stratified by IPI Age. Significant differences in expression between groups are marked with asterisks (*P < 0.05, **P < 0.01), while “ns” indicates no significant difference

Based on clinical data, cognitive impairments were categorized into language disorders, memory impairments, and executive function impairments. We computed DEGs for both the disease and control groups and performed enrichment analyses on these DEGs. In the language disorder group, we identified 78 DEGs, comprising 23 upregulated genes and 55 downregulated genes (Fig. 10A and B). These genes were found to be involved in cytokine-cytokine receptor interactions, cell adhesion molecules, and hematopoietic cell lineages (Fig. 11A). In the memory impairment group, we identified 67 DEGs, including 47 upregulated and 20 downregulated genes (Fig. 10C and D). These genes play significant roles in the NF-κB signaling pathway, MAPK signaling pathway, and apoptosis (Fig. 11B). In the executive function impairments group, we identified 545 DEGs, consisting of 310 upregulated and 235 downregulated genes (Fig. 10E and F). These genes were notably involved in the MAPK signaling pathway (Fig. 11C).

The DEGs identified under language, memory, and executive function impairments shared one common gene: ZNF619. RGS1 and DUSP1 showed differential expression across all three cognitive impairments (Fig. 11D). To further explore the association between these DEGs and m6A-related genes, we performed protein interaction analysis using the STRING database to examine the interaction network. We eliminated isolated nodes to construct the PPI network and used CytoHubba to identify core factors. The core factor network revealed that BRCA1 and CD74 had the closest relationships with other proteins. Additionally, the m6A-related genes RPL14 and PARP1 also exhibited strong associations with other proteins (Fig. 11E). We conducted enrichment analysis on the top twenty interacting genes within the network, which revealed significant roles in the TNF signaling pathway and leukocyte transendothelial migration (Fig. 11F).

### Association of m6A-related genes with clinical and pathological features in epilepsy patients

We analyzed the expression levels of m6A-related genes in relation to various clinical and pathological factors in an epilepsy cohort, including sex, age at surgery, Initial Presentation injury (IPI) age, and age of epilepsy onset. Samples were grouped based on the median IPI age to assess changes in gene expression between high and low groups. The results indicated that LSR, PARP1, and ZNF619 exhibited higher expression levels in the high group (Fig. 11G).

## Discussion

In this study, we explored the role of m6A in neurological diseases by integrating multi-omics data, including large-scale GWAS, m6A-QTL, eQTL, and pQTL datasets. Through this approach, we identified significant associations between m6A sites and three diseases: epilepsy, ischemic stroke, and VaD. We initially detected 232 associations involving 218 m6A sites linked to these conditions and subsequently found 3,430 associations between m6A sites and gene expression, implicating 2,457 genes. Further analysis revealed 3,224 associations between gene loci and the three diseases, identifying 79 genes shared between epilepsy and ischemic stroke, 59 genes between epilepsy and VaD, and 88 genes between ischemic stroke and VaD. Notably, four genes (PMF1, USP5, DYNC2LI1, and CENPW) were common to all three diseases. By integrating these findings, we identified 81 “m6A-gene-disease” associations that suggest potential mechanisms by which m6A may influence disease progression through gene regulation. Pathway enrichment analysis revealed that genes associated with each disease were involved in distinct biological processes: epilepsy-related genes were linked to blood-brain barrier function, ischemic stroke-associated genes were involved in immune responses, and VaD-related genes were implicated in RNA stability and apoptosis. Additionally, our MR analysis identified causal relationships between specific genes and disease outcomes, such as NBL1 in epilepsy, TPGS2 in ischemic stroke, and SERINC2 in VaD. Further, we conducted PPI network analysis on significant genes, identifying core networks involving PARP1 for epilepsy, MCL1 for VaD, and connections between these and other proteins associated with ischemic stroke. This analysis supports the role of m6A in modulating gene and protein expression, influencing disease-related molecular pathways. Through this multi-omics integration, we provide insights into the regulatory roles of m6A in neurological diseases, highlighting its potential as a biomarker and therapeutic target across epilepsy, ischemic stroke, and VaD.

Our study provides insights into the genetic and epigenetic underpinnings of epilepsy, ischemic stroke, and VaD, with a particular focus on m6A RNA modifications. The observed disparities in single nucleotide polymorphism (SNP) densities among these diseases suggest distinct genetic architectures, potentially reflecting varying degrees of polygenicity. This aligns with existing literature indicating that complex neurological disorders often involve a broad spectrum of genetic variants [27]. The upward deviations in the QQ plots further support the presence of genuine association signals, underscoring the robustness of our GWAS findings.

The widespread genetic associations we observe between m6A loci and three distinct neurological conditions point to a unifying epitranscriptomic layer of regulation in neurovascular and neurodevelopmental disease. Because m6A can fine-tune mRNA fate—by altering transcript stability, splicing decisions, or translation efficiency—variants that perturb writer, eraser, or reader activity may exert broad effects on neural homeostasis. Our finding of overlapping m6A sites in epilepsy, stroke, and VaD suggests these disorders share common molecular vulnerabilities, such as dysregulated inflammation, impaired DNA repair, or altered synaptic plasticity. This is consistent with emerging work showing that age- and stress-related shifts in m6A deposition—mediated by enzymes like METTL3 and FTO—can influence neuronal survival and cognitive function. While our study highlights epigenetic mechanisms as promising therapeutic targets, we note that these in silico associations require direct experimental validation (e.g., site-specific m6A mapping and functional assays) before clinical translation [30, 31].

Our FUSION analyses further reveal the broad impact of m6A on transcriptomes: each methylation site influences, on average, over three genes, reflecting m6A’s capacity to coordinate complex gene networks. This finding dovetails with reports that brain m6A landscapes are dynamically remodeled during learning, neurogenesis, and in neurodegeneration [32–34]. Our analyses pinpoint specific m6A sites that preferentially regulate genes linked to ischemic stroke and VaD, implicating pathways of vascular integrity and metabolic homeostasis rather than canonical neurodegenerative mechanisms. This pattern suggests that, although m6A exerts broad control over neuronal transcripts, individual methylation events may engage discrete, disease-relevant molecular routes.

Our gene-disease association analysis revealed thousands of significant gene associations across the three diseases, with several genes common to all conditions, including PMF1, USP5, DYNC2LI1, and CENPW. PMF1 regulates polyamine synthesis and mitosis, forming complexes to modulate antioxidant responses under oxidative stress [35]. USP5 is a deubiquitinating enzyme essential for ubiquitin recycling, maintaining protein homeostasis, and influencing pathways related to cell survival and neurodegeneration [36]. DYNC2LI1 functions in the dynein motor complex, crucial for retrograde transport in cilia, with implications for ciliopathies affecting development and organ function [37]. CENPW is a key centromeric protein necessary for chromosome segregation during cell division and genomic stability [38]. Collectively, these genes underscore their importance in maintaining cellular integrity and function across diverse biological systems. However, these four genes have not been previously studied in the context of these three diseases. Our study not only identifies the role of these genes individually in each disease but also highlights their shared involvement across epilepsy, VaD, and stroke. This discovery opens new avenues for understanding common molecular mechanisms and potential therapeutic targets for these neurological disorders.

Our enrichment analysis reveals a tissue-specific expression pattern where ischemic stroke-related genes are significantly downregulated across multiple tissues, including blood and brain, while VaD-related genes show downregulation primarily in blood. This suggests that m6A modifications may exert differential effects based on disease context and tissue type, contributing to the unique pathophysiology observed in each condition. Recent studies highlight similar findings, where m6A modifications have been shown to play tissue-specific roles in neuroinflammation and neurodegeneration [39]. These observations align with the concept that m6A’s influence on RNA stability and translation efficiency can vary across different tissues, impacting disease progression in a context-dependent manner. Additionally, specific m6A loci, such as chr1_32377212_32377386, were identified as interacting with genes implicated in VaD, notably SERINC2 and ZCCHC17. This finding illustrates the potential for m6A modifications to mediate disease-specific gene regulatory pathways. Recent research has increasingly demonstrated that m6A-modified RNAs can selectively interact with binding proteins and impact cellular processes like RNA nuclear export, stability, and splicing in a disease-specific manner [40, 41]. In the context of VaD, m6A modifications may influence the stability and expression of critical genes involved in neurovascular integrity and inflammatory response, thereby modulating disease outcomes [13]. These findings underscore the importance of m6A in shaping gene expression landscapes relevant to neurological disease progression.

Additionally, in epilepsy, using genes identified through m6A-gene-disease associations for MR analysis. We found that RP11-267M23.4, NBL1, ENTPD3-AS, RNF157, ZNF619, RPL14, and PARP1 showed causal relationship to epilepsy. Among all exposure genes, RP11-267M23.4, ENTPD3-AS, ZNF619, and RPL14 were demonstrated as risk factor to epilepsy, while NBL1, RNF157, and PARP1 were protective factors to epilepsy. These genes are involved in diverse biological processes and are often studied in various research fields. For instance, RP11-267M23.4 is a long non-coding RNA generally associated with gene expression regulation [42]. ENTPD3-AS likely regulates its neighboring gene ENTPD3, which is involved in immune response and neural signaling [43]. ZNF619 belongs to the zinc finger protein family, commonly studied for its role in gene transcription regulation, often related to cancer and immune diseases [42]. RPL14 is a ribosomal protein, crucial for protein synthesis and often linked to cancer and developmental disorders [44]. NBL1 is a secreted protein with roles in cell proliferation and differentiation, frequently researched in neuroblastoma and tumor biology [45]. RNF157, part of the E3 ubiquitin ligase family, is essential for protein degradation and has implications in neurodegenerative diseases and cancer [46]. A recent study found that a mutation in the KIF4 protein enhances its binding to PARP1, leading to a suppression of PARP1 activity. This reduction in PARP1 activity disrupts the TrkB-KCC2 pathway, alters dendritic branching and spine morphology, and impairs intracellular chloride homeostasis in neurons, which collectively increase the susceptibility to seizures. Therefore, while PARP1 plays a neuroprotective role under normal conditions, its reduced activity due to KIF4 mutation becomes a risk factor for epilepsy, highlighting PARP1 as a potential therapeutic target for antiepileptic interventions. This finding aligns closely with our own results. Through MR, we observed an odds ratio of 0.738 with *p* < 0.05, indicating that PARP1 also functions as a protective factor [47]. Our results reinforce the notion that maintaining normal PARP1 activity is crucial for reducing seizure susceptibility, supporting its potential as a therapeutic target in antiepileptic strategies.

In ischemic stroke, we found that MPO, CALCRL, PPP5C, KTN1-AS1, CCR1, CTB-50L17.9, CCR9, ZNF362, ZIK1, ELP5, CKAP2, NUP88, and SEC16A show risk effects (OR > 1). In contrast, TPGS2, ZNF683, SCAND1, ZNF362, CD40, and FBXL12 exhibit protective effects (OR < 1). MPO is an enzyme involved in oxidative stress and inflammation, with elevated levels linked to an increased risk of ischemic stroke due to its role in promoting oxidative damage and vascular inflammation [48]. CALCRL is a protein-coding gene associated with vascular functions; changes in its expression may affect vascular integrity and function, potentially influencing stroke risk [49]. CCR1 is a chemokine receptor involved in inflammatory responses, and its activation can contribute to vascular inflammation, a known risk factor for ischemic stroke. CCR9 plays a role in immune cell migration and inflammation; while primarily related to gut immunity, systemic inflammation involving CCR9 may impact vascular health and stroke susceptibility [50]. CD40 is a costimulatory protein on antigen-presenting cells that is essential for immune responses, and its role in inflammatory pathways suggests a potential link to ischemic stroke, as inflammation is a critical component in stroke pathophysiology [51]. The genes that currently lack a direct association with ischemic stroke include PPP5C, KTN1-AS1, CTB-50L17.9, ZNF362, ZIK1, ELP5, CKAP2, NUP88, SEC16A, TPGS2, ZNF683, SCAND1, and FBXL12. These genes are primarily involved in diverse cellular functions such as transcriptional regulation, protein trafficking, immune cell differentiation, microtubule stability, and protein degradation. They have been more frequently studied in the context of cancer, immune response, cell division, and neurodevelopment, but current research does not link them specifically to ischemic stroke. Further studies may reveal more about their potential indirect or broader roles in ischemic stroke. In VaD, we identified several genes associated with VaD, categorizing them as risk or protective factors. RP11-397E7.4, MCL1, SERINC2, and SMPD2 were identified as risk factors, suggesting their higher expression may increase the risk of VaD. In contrast, MRPL45P2 and EME2 appeared to be protective factors, indicating a potential role in reducing risk. Despite their significance in various biological processes, including apoptosis regulation (MCL1), mitochondrial function (MRPL45P2), and lipid metabolism (SERINC2 and SMPD2), there is currently insufficient evidence directly linking these genes to VaD [42]. Further studies are needed to clarify their roles in this condition.

The PPI network analysis revealed significant associations between PARP1, MCL1, and CD40, highlighting potential common pathways involved in ischemic stroke, epilepsy, and VaD. PARP1, a key player in DNA repair and cellular stress response, interacts with MCL1, an anti-apoptotic protein implicated in neuronal survival and inflammation. The interaction between PARP1 and MCL1 suggests that PARP1 may regulate cell survival pathways, potentially influencing the neuroinflammatory processes observed in neurovascular diseases, including ischemic stroke and VaD [52, 53]. Furthermore, the interaction between MCL1 and CD40—a co-stimulatory protein involved in immune responses—indicates a link between apoptotic regulation and immune modulation in the context of ischemic stroke [54]. CD40 activation has been shown to contribute to inflammation, which is a key factor in the pathogenesis of ischemic stroke [55]. Thus, the PARP1-MCL1-CD40 axis may play a critical role in modulating neuroinflammation, apoptosis, and cellular repair mechanisms, which are central to the progression of ischemic stroke, VaD, and other neurodegenerative conditions. This network suggests that targeting the interactions within this pathway could offer therapeutic potential for diseases involving vascular and neuroinflammatory damage. Further research is needed to clarify the functional significance of these interactions and their role in disease pathophysiology.

Our pQTL-based Fusion analysis uncovered 344 proteins with 371 disease associations, underscoring the proteomic dimension of m6A’s impact. Ischemic stroke yielded the most links (133), followed by VaD (123) and epilepsy (115). Notably, we found eight proteins shared by stroke and epilepsy (e.g., SH3BGRL3, PTPMT1), nine shared by epilepsy and VaD (e.g., DOCK7, BCAN), and ten by stroke and VaD (e.g., MTHFR, UVRAG), suggesting common pathogenic axes like inflammation and cellular stress. These overlapping proteins represent promising biomarkers or targets for interventions spanning multiple neurovascular conditions, though their precise functions warrant dedicated follow-up. In epilepsy, mapping our m6A-associated genes (e.g., PARP1, NBL1, RPL14) to their proteomic counterparts identified causal links to proteins such as ST6GALNAC5, CTNNA2, and TOM1L1, highlighting specific m6A-driven regulatory cascades at the protein level.

Our PPI network of DEGs stratified by cognitive domain revealed subtype-specific and shared molecular signatures: language impairments mapped to cytokine-receptor interactions and cell-adhesion pathways, memory deficits to NF-κB and MAPK signaling, and executive dysfunction again to MAPK cascades. Across all three groups, ZNF619, RGS1, and DUSP1 emerged as common nodes, while BRCA1 and CD74 ranked highest by connectivity. Notably, m6A-associated regulators RPL14 and PARP1 formed extensive links to these hubs [56, 57]. Additionally, RGS1 and DUSP1 are involved in inflammation and immune signaling, which are increasingly recognized as key contributors to cognitive decline [58]. The enrichment of TNF signaling and leukocyte transendothelial migration further implicates neuroinflammatory mechanisms. Together, these findings suggest that m6A-driven modulation of immune, inflammatory, and synaptic pathways contributes to cognitive impairment and nominate these proteins as candidates for biomarkers or targeted intervention.

In our epilepsy cohort, stratifying patients by median IPI age revealed that LSR, PARP1, and ZNF619 are significantly upregulated in those with later-onset or more severe presentations, suggesting that m6A regulation of lipid metabolism, DNA repair/stress response, and synaptic plasticity may modulate disease trajectory. While LSR’s role in neuroinflammation, PARP1’s involvement in neuronal survival, and ZNF619’s regulation of transcription are consistent with epilepsy pathophysiology, these associations are based on a limited sample size and bulk‐tissue expression [59]. PARP1, a gene involved in DNA repair and stress responses, could influence neuronal survival and excitability in epilepsy [60]. Similarly, ZNF619, a zinc-finger protein, may be involved in regulating synaptic functions and neuronal plasticity, further suggesting its relevance in epilepsy [61]. These results highlight that m6A-related genes such as LSR, PARP1, and ZNF619 may play important roles in the clinical and pathological features of epilepsy, particularly in relation to disease onset and progression. As such, they should be viewed as hypothesis‐generating; targeted validation in larger, cell‐type–specific cohorts and functional assays will be essential to confirm their utility as biomarkers or therapeutic targets.

We also recognize that m6A regulation is highly cell-type specific within the brain and that dissecting neuron versus glia methylation landscapes could yield critical insights into disease mechanisms. Neurons rely on dynamic m6A “writing” and “reading” to fine-tune synaptic plasticity and memory consolidation, whereas glial cells leverage m6A to control inflammatory and repair programs; dysregulation in either compartment may therefore contribute to neurodegeneration along distinct axes. For example, altered METTL3 or YTHDF2 activity in excitatory neurons has been shown to impair long-term potentiation and accelerate cognitive decline, while increased FTO-mediated demethylation in microglia promotes pro-inflammatory cytokine production and blood-brain barrier disruption. As single-cell m6A-RIP and RNA-seq methods mature, mapping these cell-type-specific methylomes will be essential to understand how neuron-intrinsic versus glia-driven m6A alterations converge on common pathways of oxidative stress, synaptic loss, and neuroinflammation in epilepsy, stroke, and dementia. We look forward to integrating these emerging datasets to refine our multi-omic framework and pinpoint cell-type-restricted therapeutic targets.

We note that m6A has been shown to decorate key regulators of synaptic function and cognition—such as BDNF, CAMK2A, GRIN1, ARC, and FMR1—modulating their mRNA stability and local translation at dendrites to control long-term potentiation and memory consolidation. We further note that several of our top m6A-associated genes (e.g. PARP1, RPL14, ZNF619) intersect with pathways downstream of these canonical synaptic plasticity players, suggesting that altered methylation of both core plasticity transcripts and their regulators may converge to influence seizure susceptibility and cognitive outcomes.

Several limitations should be considered in this study. For instance, the sample sizes for some neurological diseases, such as epilepsy, ischemic stroke, and VaD, as well as for the m6A datasets, may be relatively small, potentially limiting the power to detect significant associations. Larger sample sizes are required to confirm our findings and identify additional loci that may be involved in disease pathogenesis. Furthermore, the use of FUSION and SMR & HEIDI tools to separately identify m6A-disease, m6A-gene, and gene-disease associations could result in a loss of power due to the thresholding of results based on P-values at multiple stages. A key direction for future research is to develop a method that integrates GWAS data with all omics data in a single, more unified analysis, which would improve the precision and robustness of the results.

In conclusion, this study successfully identified the relationship between m6A modifications and multiple molecular phenotypes in neurological diseases, including epilepsy, ischemic stroke, and VaD. We discovered m6A-related sites and associated genes for these conditions, highlighting potential functional pathways through responses to stimuli. These findings support the genetic involvement of m6A in neuropsychiatric disorders and emphasize the complex interplay between multiple molecular phenotypes. Additionally, our study provides promising targets for further mechanistic investigations and the development of novel therapeutic strategies aimed at modulating m6A pathways in neurological diseases.

## Electronic supplementary material

Below is the link to the electronic supplementary material.

Supplementary Material 1

Supplementary Material 2

Supplementary Material 3

Supplementary Material 4

Supplementary Material 5

Supplementary Material 6

Supplementary Material 7

## Data Availability

No datasets were generated or analysed during the current study.

## Abbreviations

m6A-N6: Methyladenosine
VaD: Vascular Dementia
GWAS: Genome-Wide Association Study
TWAS: Transcriptome-Wide Association Study
SMR: Summary-data-based Mendelian Randomization
QTL: Quantitative Trait Loci
eQTL: Expression Quantitative Trait Loci
pQTL: Protein Quantitative Trait Loci
DEGs: Differentially Expressed Genes
KEGG: Kyoto Encyclopedia of Genes and Genomes
GO: Gene Ontology
MCAO: Middle Cerebral Artery Occlusion
SNP: Single Nucleotide Polymorphism
QQ Plot: Quantile-Quantile Plot
LD: Linkage Disequilibrium
IVW: Inverse Variance Weighting
MR: Egger-Mendelian Randomization-Egger Regression
FDR: False Discovery Rate
IPI: Initial Presentation Injury
TNF-α: Tumor Necrosis Factor Alpha
IL-1: Interleukin 1
PARP1: Poly (ADP-Ribose) Polymerase 1
MCL1: Myeloid Cell Leukemia 1
CCR1: C-C Chemokine Receptor Type 1
CCR9: C-C Chemokine Receptor Type 9
