# Supplementary material for: Multi-omics integration reveals the role of N6-methyladenosine in epilepsy, ischemic stroke, and vascular dementia
Source: Mol Brain. 2025 Jul 7;18:58. doi: 10.1186/s13041-025-01228-4 (PMC12236031; doi:10.1186/s13041-025-01228-4)
Supplement: Supplementary file 7 — Supplementary Material 7 [file 13041_2025_1228_MOESM7_ESM.docx]

**Table S1: Association of m6A with three diseases.**

Table S1 summarizes the significant associations between 218 m6A methylation sites and three neurological diseases: epilepsy, ischemic stroke, and vascular dementia (VaD). A total of 232 associations were identified, with ischemic stroke showing the highest number (96 associations), followed by epilepsy and VaD (68 associations each). The table highlights overlaps in m6A sites across diseases, including 8 shared sites between epilepsy and ischemic stroke, 1 site between epilepsy and VaD, and 5 sites between ischemic stroke and VaD.

**Table S2: m6A Sites and Gene Expression Associations.**

Table S2 presents 3,430 significant associations between 790 m6A sites and 2,457 genes, identified using the FUSION pipeline. All associations passed multiple testing correction (P < 0.05). The data demonstrate that m6A sites regulate gene expression in brain tissue, with each site influencing approximately 3.11 genes on average.

**Table S3: Gene Loci Associated with Diseases.**

Table S3 lists 3,224 significant associations involving 3,979 genes across epilepsy, ischemic stroke, and VaD. Ischemic stroke exhibited the most gene associations (1,242), followed by VaD (1,054) and epilepsy (928). The table highlights overlaps, including 79 genes shared between epilepsy and ischemic stroke, 59 between epilepsy and VaD, and 88 between ischemic stroke and VaD. Four genes (PMF1, USP5, DYNC2LI1, CENPW) were common to all three diseases.

**Table S4: m6A-Gene-Disease Associations.**

Table S4 integrates m6A-disease and gene-disease associations to reveal key regulatory networks. Epilepsy is linked to 13 genes and 9 m6A sites, ischemic stroke to 52 genes and 31 m6A sites, and VaD to 17 genes and 15 m6A sites. This integration illustrates how m6A modifications influence gene expression and disease outcomes.

**Table S5: Mendelian Randomization (MR) Analysis Results.**

Table S5 summarizes MR analysis results identifying causal relationships between gene expression and disease outcomes. Key findings include NBL1 (epilepsy, OR=0.722), TPGS2 (ischemic stroke, OR=0.891), and SERINC2 (VaD, OR=1.193). Methods included inverse-variance weighted (IVW) regression and sensitivity analyses, confirming robustness of associations.

**Table S6: Protein Associations with Diseases.**

Table S6 details 371 significant associations between 344 proteins and the three diseases. Ischemic stroke showed the highest protein associations (133), followed by VaD (123) and epilepsy (115). Overlaps include 8 proteins shared between epilepsy and ischemic stroke, 9 between epilepsy and VaD, and 10 between ischemic stroke and VaD.
